# Replication stress-induced alternative mRNA splicing alters properties of the histone RNA-binding protein HBP/SLBP: a key factor in the control of histone gene expression

**DOI:** 10.1042/BSR20130074

**Published:** 2013-09-06

**Authors:** Alexander M. J. Rattray, Pamela Nicholson, Berndt Müller

**Affiliations:** *School of Medical Sciences, University of Aberdeen, Institute of Medical Sciences, Foresterhill, Aberdeen AB25 2ZD, Scotland, U.K.; †Department for Chemistry and Biochemistry, University of Bern, Freiestrasse 3, CH-3012 Bern, Switzerland

**Keywords:** alternative splicing, cellular localization, checkpoints, histone gene expression, mRNA translation, RNA-binding protein., ATM, ataxia telangiectasia mutated- and Rad3-related, bp, base pairs, CBP80/20, cap-binding protein 80/20, CTIF, CBP80/20-dependent translation initiation factor, DAPI, 4′,6-diamidino-2-phenylindole, eIF, eukaryotic initiation factor, GAPDH, glyceraldehyde-3-phosphate dehydrogenase, GFP, green fluorescent protein, HBP, hairpin-binding protein, HU, hydroxyurea, kb, kilobase, MIF1GD, MIF4G domain, nt, nucleotide, ORF, open reading frame, PABP, poly(A) binding protein, PAIP1, polyadenylate-binding protein-interacting protein 1, QPCR, quantitative PCR, RBD, RNA-binding domain, SLBP, stem-loop binding protein, SLIP1, SLBP interacting protein 1, snRNP, small nuclear ribonucleoprotein.

## Abstract

Animal replication-dependent histone genes produce histone proteins for the packaging of newly replicated genomic DNA. The expression of these histone genes occurs during S phase and is linked to DNA replication via S-phase checkpoints. The histone RNA-binding protein HBP/SLBP (hairpin-binding protein/stem-loop binding protein), an essential regulator of histone gene expression, binds to the conserved hairpin structure located in the 3′UTR (untranslated region) of histone mRNA and participates in histone pre-mRNA processing, translation and histone mRNA degradation. Here, we report the accumulation of alternatively spliced HBP/SLBP transcripts lacking exons 2 and/or 3 in HeLa cells exposed to replication stress. We also detected a shorter HBP/SLBP protein isoform under these conditions that can be accounted for by alternative splicing of HBP/SLBP mRNA. HBP/SLBP mRNA alternative splicing returned to low levels again upon removal of replication stress and was abrogated by caffeine, suggesting the involvement of checkpoint kinases. Analysis of HBP/SLBP cellular localization using GFP (green fluorescent protein) fusion proteins revealed that HBP/SLBP protein and isoforms lacking the domains encoded by exon 2 and exons 2 and 3 were found in the nucleus and cytoplasm, whereas HBP/SLBP lacking the domain encoded by exon 3 was predominantly localised to the nucleus. This isoform lacks the conserved region important for protein–protein interaction with the CTIF [CBP80/20 (cap-binding protein 80/20)]-dependent initiation translation factor and the eIF4E (eukaryotic initiation factor 4E)-dependent translation factor SLIP1/MIF4GD (SLBP-interacting protein 1/MIF4G domain). Consistent with this, we have previously demonstrated that this region is required for the function of HBP/SLBP in cap-dependent translation. In conclusion, alternative splicing allows the synthesis of HBP/SLBP isoforms with different properties that may be important for regulating HBP/SLBP functions during replication stress.

## INTRODUCTION

Eukaryotic chromatin is composed of DNA and histone proteins. The correct chromatin structure is essential for cell division and gene expression. In animals, the chromatin structure is maintained during DNA replication by S-phase-limited expression of the replication-dependent histone genes (from here on referred to simply as ‘histone’ genes). Animal histone genes produce mRNAs that are not polyadenylated; instead they contain a highly conserved hairpin structure at their 3′ ends.

Histone proteins are only produced during S phase of the cell cycle. The expression of histone genes is co-ordinately regulated and finely balanced with DNA replication and disturbing this balance can severely affect genomic stability and gene expression by inducing DNA damage and replication fork collapse [1,2]. At the level of RNA, three major processes: transcription, mRNA 3′ end processing and mRNA stability control contribute to the complex regulation of histone gene expression during S phase [3,4]. This combination of transcriptional and post-transcriptional control acts to regulate the 35-fold increase of histone mRNA levels as cells progress from G1 into S phase and ensures that the mRNA levels return to baseline levels as the cells exit S phase [5].

In metazoans, the majority of this cell cycle regulation is carried out post-transcriptionally and is predominantly mediated by the conserved RNA hairpin element and its *trans*-acting binding partner the HBP/SLBP (hairpin-binding protein/stem-loop-binding protein) [6,7]. The gene-encoding human HBP/SLBP is located on chromosome 4 (4p16.3) and has an 810 nt (nucleotide) ORF (open reading frame) distributed over eight exons, which generates a 270 amino acid protein [8].

HBP/SLBP expression itself is also cell cycle regulated. In human cells, the intracellular concentration of HBP/SLBP in cycling cells is not detectable in G1 phase but begins to accumulate at the G1/S phase transition and remains at a high level during S phase [9]. At the end of S phase, it is degraded by the proteasome pathway and is not detectable again until the G1/S phase boundary. Therefore it accumulates and is degraded in parallel to histone mRNAs. It has been suggested that the increase in HBP/SLBP abundance is because of a combination of a 2-fold increase in transcription rate combined with an increase in the rate of translation in S phase, but there is no direct evidence for this. The degradation at the end of S phase is thought to be triggered by protein phosphorylation at several sites including the Thr^61^Thr^62^Pro^63^ motif in the N-terminal domain of HBP and occurs by the ubiquitin–proteasome pathway [10,11]. The degradation of HBP/SLBP is temporally linked to the disappearance of histone mRNAs from the cell. The absence of HBP/SLBP outside S phase prevents 3′ end processing of histone pre-mRNAs and restricts the generation of mature histone transcripts to S phase. However, histone mRNA is still destabilised upon exit from S phase even in cells that cannot degrade HBP/SLBP due to exposure to a proteasome inhibitor or expression of a stabilised HBP/SLBP protein [12]. Despite the fact that in such experiments HBP/SLBP was generally expressed from a plasmid, these findings are compatible with there being no direct link between HBP/SLBP levels and histone mRNA stability reported to date. Localization studies have shown that HBP/SLBP can be found in both the nucleus and the cytoplasm, consistent with its known roles in histone pre-mRNA processing in the nucleus and their subsequent translation in the cytoplasm [13].

HBP/SLBP binds to histone pre-mRNA in the nucleus and participates in histone mRNA 3′ end processing. It undergoes interactions with ZFP100, a component of the U7 snRNP (small nuclear ribonucleoprotein); another factor involved in histone RNA 3′ end processing that determines the position of the cleavage site [14,15]. The RNA CPSF (cleavage and polyadenylation specificity factor)-73 is recruited to the cleavage site by association with the U7snRNP [16]. After processing, the mature histone transcripts are exported to the cytoplasm with HBP/SLBP still bound to the hairpin element at the 3′ end. The histone RNA hairpin element has been shown to be important for nuclear export [17,18], but any involvement of HBP/SLBP in this process is less clear. Using *Xenopus laevis* oocytes as an experimental system, it was found that histone mRNA export was independent of SLBP, but a more recent report utilizing an artificial nuclear export assay in human cells suggests that human HBP/SLBP may be involved in this process [19,20].

In the cytoplasm, HBP/SLBP stimulates translation of histone mRNAs [21–23]. HBP/SLBP is thought to interact with a histone mRNA specific translation factor called SLIP1 (SLBP-interacting protein 1, or MIF4GD (MIF4G domain)-containing protein) as well as with eIF3 (eukaryotic initiation factor 3) and PAIP1 (polyadenylate-binding protein-interacting protein 1) [21,22,24]. It has been proposed that HBP/SLBP binds SLIP1 that in turn binds the 5′ cap-binding protein, eIF4E and hence circularises the mRNA. SLIP1 binds to *Xenopus* SLBP1 via a conserved 15 amino acid region known to be critical for activation of histone mRNA translation [21,23]. SLIP1 also interacts with eIF4G, possibly in the same region as the PABP (poly(A) binding protein) binds [21], indicating that SLIP1 promotes translational efficiency in much the same way that PABP acts in the translation of polyadenylated transcripts. Lately, human HBP/SLBP was found to interact with the CTIF [CBP80/20 (cap-binding protein 80/20)-dependent initiation translation factor)] [25]. CTIF is involved in the first, pioneer round of translation, taking place directly after nuclear export of mRNA [25,26]. The conserved region found to be important for translation in *Xenopus* SLBP1 is also implicated in the interaction with CTIF.

The synthesis of histones and the production of DNA are intimately coupled and disrupting either histone production or DNA replication results in the inhibition of the other process. It is very likely that the signals involved in linking histone gene expression and DNA replication act at various levels to ensure they are tightly regulated. Some of this complex coupling is mediated by a poorly understood checkpoint involving the protein kinases ATR [ATM (ataxia telangiectasia mutated)- and Rad3-related] and DNA-PK (DNA-dependent protein kinase) [27,28]. This includes the rapid degradation of histone mRNAs induced under replication stress conditions. For a long time it was believed that HBP/SLBP was not involved in the stability control of histone mRNAs because while histone mRNAs rapidly disappear when replication is inhibited, HBP/SLBP remains stable until the end of S phase. Indeed, some of these factors have been shown to be required for histone mRNA decay [29]. In addition, the expression of HBP/SLBP unable to undergo an interaction with CTIF delayed the replication stress-induced decay of histone mRNA, implicating CBP80/20-mediated translation in histone mRNA decay [25]. Other factors involved in histone mRNA degradation following DNA replication inhibition include LSM1 and UPF1 [27,29] and the latter has been linked to DNA replication and to various nuclear events independent of its role in nonsense-mediated mRNA decay [30].

Here we report the identification and characterization of alternative HBP/SLBP mRNA splice variants that can be detected at the RNA and protein level under replication stress conditions. The stress-induced alternative splicing affected exons coding for domains with important functions in histone mRNA translation and HBP/SLBP localization and stability. We observed that this increase in alternative splicing under replication stress conditions was abrogated by the checkpoint kinase inhibitor caffeine. We also analysed the effect of alternative splicing on the cellular localization of HBP/SLBP isoforms using GFP (green fluorescent protein) fusion proteins. Our observations indicate that alternative splicing of HBP/SLBP mRNA may contribute to the regulation of histone gene expression; for example, during replication stress conditions by allowing for the synthesis of HBP/SLBP proteins with tailored functional properties.

## MATERIALS AND METHODS

### Cell culture

HeLa cells were grown in DMEM (Dulbecco's modified Eagle's medium) supplemented with 10% (v/v) FBS (fetal bovine serum) at 37°C in a 5% (v/v) CO_2_ atmosphere. Cells were synchronised at G1/S phase transition by double thymidine block. Treatment with HU (hydroxyurea) was at 2 mM. Treatment with 5 mM caffeine (Sigma-Aldrich) for 1 h prior to the addition of HU was performed as previously described [28].

### Plasmids

A HBP/SLBP fragment containing the HBP/SLBP ORF immediately preceded by an EcoRI site was released from pCI-neo-N-HA [31,32] by restriction with EcoRI and DraI and inserted into pEGFP-C2 (Clontech) cleaved with EcoRI and SmaI. Exons 2 and/or 3 were deleted from this construct by site-directed mutagenesis using the QuikChange Lightning mutagenesis kit (Agilent Technologies) with primer CGACGGTGACGCCAGCTTTACCACTCCTGAAGGCCC and its reverse complement for deletion of exon 2; primer GTCATTGATGAGGAGTTTCCTTTTATATCTCTCGGGTCTGCGCTC and its reverse complement for deletion of exon 3; primer GCGACGGTGACGCCAGATATAAAAGGAAACTCCTCATCAATGACTTTG and its reverse complement for deletion of exons 2 and 3. All constructs were analysed by DNA sequencing (DNA Sequencing & Services, Dundee University).

### Analysis of alternative splicing of HBP/SLBP mRNA by semi-QPCR (quantitative PCR)

RNA was prepared by extraction with TRIzol (Life Technologies). Normally, 2 μg total RNA was reverse transcribed using random primers and MMLV (Moloney murine leukaemia virus) RT (reverse transcriptase) (Promega) according to the manufacturer's instructions. Alternative splicing of HBP/SLBP was detected by PCR using primers CCGCCGAGGCATCAGAGC (primer binding site in exon 1) and AAGCTTCCAAATCAAACTCATCCTCCAC (primer binding site in exon 8) and GoTaq DNA polymerase (Promega). PCR reactions were performed as recommended by the manufacturer and cycling conditions were 5 min at 94°C, followed by 40 cycles of 30 s at 94°C, 30 s at 64°C and 1 min at 72°C, followed by a final 10 min at 72°C. PCR reactions were analysed by agarose gel electrophoresis and the DNA was detected by ethidium bromide staining. Images were captured and analysed using Gene Tools (Syngene). The frequency of alternative splicing was calculated without compensating for the different lengths of the fragments. To confirm that the bands represent alternative spliced forms of HBP/SLBP mRNA, the PCR products were isolated, cloned into pGEM-T Easy (Promega) and sequenced (DNA Sequencing & Services, Dundee University).

To detect alternative splicing of β-tubulin mRNA (NM_178014.2), primers GGTGCCAAGTTCTGGGAGGTG and GGAAACGGAGGCAGGTGGTG were used for PCR. Primer binding sites are located to sequences in the first and last exon (exon 4) of this transcript, and a 679 bp (base pairs) PCR product contains all exons. To detect alternative splicing of GAPDH (glyceraldehyde-3-phosphate dehydrogenase) mRNA (NM_002046.3), primers TGGGGAAGGTGAAGGTCGGAG and CTCTTGCTGGGGCTGGTGG were used. Primer binding sites are located in exon 2, which contains the start of the ORF, and the last exon (exon 9). The full length PCR product spanning exons 2–9 is expected to be 1017 bp in length. ZFP100 (ZNF473) was detected using primers CGTGTGCGGGGAGTTGAATC and CCAGGTGTCCTCTATACAGGCTTCTCC. Primer binding sites are located in exons 1 and 5, which is the last exon. These primers detect ZFP100 transcript variants 1 and 2 (NM_015428.1; NM_001006656.1), and produce 631 and 509 bp PCR products, respectively. The PCR products were isolated, cloned into pGEM-T Easy (Promega) and their identity was confirmed by sequencing (DNA Sequencing & Services, Dundee University).

### SDS/PAGE and Western blotting

Cell lysates were prepared by cell lysis using RIPA buffer supplemented with protease inhibitors (Roche). Proteins (20 μg/sample), along with a pre-stained marker (ColorPlus™ Prestained Protein Ladder from New England Biolabs) were separated by SDS/12%PAGE. Proteins were transferred onto Hybond-P membrane (GE Healthcare). HBP/SLBP was detected using affinity purified anti HBP/SLBP serum. The serum was used previously [33] and was purified by binding to human His-tagged recombinant HBP/SLBP expressed in insect cells using a baculovirus protein expression system and immobilised on Hybond-N membrane (GE Healthcare). The bound antibody was eluted with 200 mM glycine, pH 2.8, and the pH of the eluate was then adjusted to pH 7.5 with 1M Tris base, and supplemented with 1% (w/v) BSA. Antibody staining was detected by chemiluminescence (ECL Plus Western Blotting Detection Reagents or kit, GE Healthcare), using HRP (horseradish peroxidase)-coupled anti-mouse or anti-rabbit secondary antibodies (Cell Signalling) and Kodak MXB films.

### Northern blotting

Northern blotting was performed as described previously [28]. HBP/SLBP mRNA was detected using a 460 bp probe spanning nts 416–876 (exons 4–8) prepared by PCR amplification from HBP/SLBP cDNA. RNA was visualized using Kodak MXB films or using a FujiFilm FLA-3000 Image Analyser and analysed using AIDA software.

### Microscopy

Plasmids expressing GFP fusion proteins were transfected into HeLa cells growing on cover slips. Cells were synchronised by double thymidine block, and transfected during the period between the first and second thymidine treatment. Following the second block, the cells were either released into S phase for 4 h or they were released for 3 h followed by a 1 h treatment with 5 mM HU to degrade histone mRNA (4 h total post-release). For visualization, 4 h after release from the second thymidine block, the cells were fixed with 4% (v/v) PFA (paraformaldehyde) and the cover slips were mounted with Vecta-Shield mounting medium containing DAPI (4′,6-diamidino-2-phenylindole) and finally analysed using a Zeiss LSM 700 laser scanning microscope with ZEN software (Zeiss). For this analysis, exposures were adjusted for background fluorescence levels using untransfected cells.

## RESULTS

### Alternative splicing of HBP/SLBP transcripts gives rise to several mRNAs and proteins

**Figure 1:**
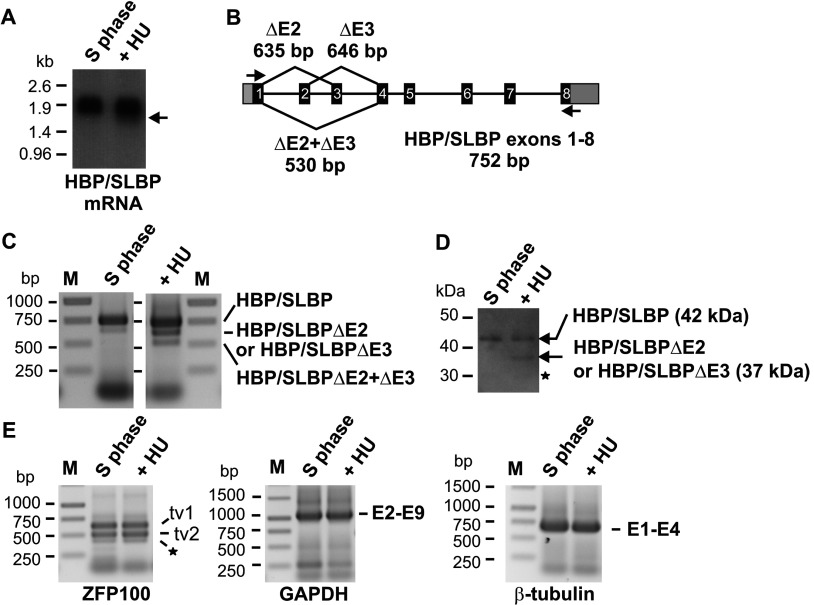
Alternative splicing of HBP/SLBP transcripts (A) Analysis of HBP/SLBP mRNA by Northern blotting. Total RNA was isolated from HeLa cells synchronised by dT (double thymidine) block and released into S phase for 6 h (S phase), and from S-phase HeLa cells released into S phase for 3 h and then treated with HU for a further 3 h (+HU). The arrow indicates HBP/SLBP RNA with increased mobility. RNA size standards are indicated in kb. (B) Schematic representation of HBP/SLBP splice variants generated by skipping of exons 2 and/or 3. The lengths of PCR products with primers (black arrows) used to detect alternative splicing are indicated. (C) Analysis of HBP/SLBP mRNA splicing by semi-quantitative PCR. RNA prepared from S phase HeLa cells and S phase HeLa cells treated for 3 h with +HU was reverse transcribed using random primers. HBP/SLBP splice variants were amplified by PCR from cDNA and analysed by agarose gel electrophoresis as described in Materials and Methods. HBP/SLBP mRNA splice variants are indicated. M indicates the molecular markers, with the size in bp. (D) Analysis of HBP/SLBP proteins. Lysates prepared from HeLa cells 6 h after release into S phase and HeLa cells released into S phase and after 3 h treated with +HU for a further 3 h were analysed by Western blotting using affinity purified HBP/SLBP antibody. ★ indicates a weak signal detected in both samples in the region where HBP/SLBPΔE2+ΔE3 would be expected. (E) Analysis of splicing of GAPDH, β-tubulin and histone processing factor ZFP100 mRNAs. mRNA splicing was analysed as described in Materials and Methods. ZFP100 tv (transcript variants) 1 and 2 were detected in both the RNA prepared from HeLa cells in S phase and from S-phase HeLa cells treated for 3 h with HU. In addition, we detected a minor variant lacking exon 2 (★). GAPDH mRNA products spanning exons 2–9 and containing the ORF, and β-tubulin mRNA spanning exons 1–4 and containing the ORF were detected in both RNA preparations. For clarity, pictures are shown in inversed colours. M indicates the molecular markers.

The RNA-binding protein HBP/SLBP is a central regulator that co-ordinates the expression of all histone genes. HBP/SLBP is essential for histone mRNA 3′ end formation and is involved in translation of histone mRNA, as well as playing a role in the control of histone mRNA stability [6,7]. A bioinformatic survey of human HBP/SLBP sequences led to the identification of two alternatively spliced variants; a variant that lacks exon 3 and one that lacks exon 2 out of a total of eight exons (Figure 1). Both sequences were present in the NCBI (National Centre for Biotechnology Information) EST database. The human variant lacking exon 3 is represented by BG614662, CN398104, DC360271, DC387327, DC424059 and HY180290 found in RNA isolated from testicular embryonal carcinoma, embryonic stem cells, neuroblastoma, embryos and mixed human RNA samples. The variant lacking exon 2 encoded by DA564172 was found in RNA isolated from heart tissue. Exons 2 and 3 encode elements found to be important for HBP/SLBP nuclear localization and degradation as well as histone mRNA translation but not for RNA binding [12,13,22,23,34] (see also Figure 4 below). Therefore alternative splicing is expected to produce proteins with altered properties. We wanted to determine whether we could detect alternative splicing of HBP/SLBP mRNA. In dividing cells, alternative splicing is rare and has not been reported. To explore whether alternative splicing may occur in cells subjected to replication stress conditions we compared HBP/SLBP mRNA structure in S-phase HeLa cells and in S-phase HeLa cells treated with the DNA replication inhibitor HU (hydroxyurea). HU is an inhibitor of ribonucleotide reductase. Treatment with this compound prevents the dNTP pool expansion that normally occurs at the G1/S phase boundary and causes replication stress conditions. Treatment with HU is also known to lead to the termination of histone gene expression in S phase cells (see e.g. [28]). RNAs were analysed by Northern blotting (Figure 1A). HBP/SLBP mRNA isolated from S-phase cells migrated as expected with the mobility of about 1.9 kb (kilobase) mRNA [8]. In RNA prepared from cells treated with HU, the majority of HBP/SLBP mRNA migrated with the same mobility but we also detected some HBP/SLBP mRNA with increased mobility shown by the arrow in Figure 1(A).

We decided to investigate whether the appearance of the faster migrating HBP/SLBP mRNA was really because of alternative splicing of HBP/SLBP mRNA. We designed a semi-quantitative PCR-based assay with PCR primers located in the first and last exon of HBP/SLBP (exons 1 and 8, respectively; Figure 1B). PCR amplification of full-length cDNA containing all eight exons produces a 752 bp fragment while alternatively spliced transcripts produce shorter PCR products. The analysis of splicing using cDNA prepared from total RNA isolated from HeLa cells in S phase, followed by PCR amplification and agarose gel electrophoresis (Figure 1C), produced predominately approximately 750 bp long fragments (90.7%) and a small proportion of approximately 640 bp long fragments (9.3%), compatible with the majority being full length HBP/SLBP transcripts and a minority of alternatively spliced transcripts, respectively. The length of the minor species of PCR products is compatible with mRNAs lacking exons 2 or 3 (635 bp and 646 bp, respectively). The identities of the PCR products were determined by cloning into pGEM-T Easy followed by Sanger sequencing analyses. This confirmed that the 750 bp products corresponded as expected to full-length HBP/SLBP transcripts, while the shorter fragment matched with the HBP/SLBP mRNA splice variant lacking exon 3. From here on, we refer to these splice variants as HBP/SLBP and HBP/SLBPΔE3, respectively.

We compared this observation to splicing occurring in cells treated with HU (Figure 1C). In RNA prepared from cells treated with HU for 3 h we detected approximately 750 bp long fragments (62.8%) and an increased amount of approximately 640 bp long fragments (25.6%) corresponding to HBP/SLBPΔE3 or possibly to HBP/SLBP lacking exon 2 (HBP/SLBPΔE2). Additionally, we detected another PCR product of about 500 bp (11.6%). Products of this length (530 bp) are expected for a transcript lacking both exons 2 and 3. The identities of these PCR products were determined by sequencing analyses. As expected, approximately 750 bp long fragments were full length HBP/SLBP mRNAs. The approximately 500 bp long fragments did correspond to the splice variant lacking both exons 2 and 3 (hereafter named HBP/SLBPΔE2+ΔE3). This sequence is also represented in the EST database (CR976286.1). Fragments of approximately 640 bp which may correspond to either HBP/SLBPΔE2 or HBP/SLBPΔE3 were also analysed. 89% of clones analysed represented HBP/SLBPΔE3 and the remaining 11.1% matched to HBP/SLBPΔE2 mRNA (*n*=18). This indicates that the majority of these approximately 640 bp long fragments represent the HBP/SLBPΔE3 splice variant mRNA. Note that our findings do not rule out that HBP/SLBPΔE2 mRNA is also present in S-phase cells, but the analysis above indicates that this would occur at a low frequency. The ORFs are maintained in HBP/SLBPΔE2, HBP/SLBPΔE3 and HBP/SLBPΔE2+ΔE3 transcripts, indicating that all these transcripts may encode HBP/SLBP protein isoforms. We tested whether these splice variants can be found in polysomes, and observed the association of HBP/SLBP, HBP/SLBPΔE2 and HBP/SLBPΔE3 transcripts and to a lesser degree of HBP/SLBPΔE2+ΔE3 transcripts with ribosomes (results not shown), suggesting that these transcripts may be translated.

Next, we established whether we could detect HBP/SLBP proteins produced from alternatively spliced HBP/SLBP transcripts. Using an affinity purified rabbit polyclonal anti-HBP/SLBP antibody raised against the very C-terminus of HBP/SLBP [33] we detected HBP/SLBP migrating as expected with the mobility of a protein of about 42 kDa molecular mass (Figure 1D) in lysate prepared from S-phase HeLa cells. This protein was also detected in lysate prepared from S-phase HeLa cells treated with HU. In addition, we observed a smaller amount of a further protein migrating with the mobility of a protein of about 37 kDa in S-phase HeLa cell lysate. The predicted mass differences between HBP/SLBP and the isoforms lacking the domains encoded by exon 2 or 3 or both exons 2 and 3, are 4.6 kDa, 4 kDa and 8.6 kDa, respectively. Thus, a mass difference of about 5 kDa is compatible with this faster migrating protein being HBP/SLBPΔE2 or HBP/SLBPΔE3, but not HBP/SLBPΔE2+ΔE3. As the majority of transcripts analysed in Figure 1(C) were the HBP/SLBPΔE3 splice variants we assume that this is the predominant isoform. However, we cannot exclude the possibility that we are actually detecting a mixture of proteins composed of both isoforms. The HBP/SLBPΔE2+ΔE3 isoform is expected to migrate with the mobility of a protein of about 33 kDa. A comparison of the samples prepared from cells treated with/without HU in Figure 1(D) indicates that any amount of HBP/SLBPΔE2+ΔE3 protein produced in HU treated cells was not significantly above the background signal also present in the protein sample from S-phase cells. This indicates that the levels of HBP/SLBPΔE2+ΔE3 protein are low, reflecting the relatively low abundance of this transcript detected (Figure 1C). In conclusion, we have detected alternative splicing of HBP/SLBP transcripts under replication stress conditions, resulting in the synthesis of additional HBP/SLBP isoforms.

Given that HBP/SLBP is predominately expressed during S phase, during which it fulfils its functions in histone gene expression, we focused our further investigations on the link between HBP/SLBP splicing and replication stress, which can be induced by treatment with HU. First, it was important to examine whether HU can cause widespread non-specific changes in mRNA splicing patterns. To this end, we examined whether treatment with HU can affect the splicing patterns of GAPDH mRNA, β-tubulin mRNA or ZFP100 (or ZNF473) mRNA, using similar PCR assays as employed for HBP/SLBP (Figure 1E), followed by sequence analysis of the PCR products. ZFP100 was included because it is specifically and directly involved in histone pre-mRNA processing and, like HBP/SLBP, it is a component of the histone pre-mRNA-processing complex [35]. The analysis of splicing in HeLa cells treated with/without HU did not identify any significant changes in the splicing pattern of these mRNAs. ZFP100 produces two major transcripts, transcript variants 1 and 2 differing in the length of exon 2. In transcript variant 2, the 5′ end of exon 2 is lacking and its length is reduced from 199 to 77 nts, presumably due to alternative splicing. The major products observed for ZFP100 were 631 bp and 509 bp long, as expected for transcript variants 1 and 2, respectively. A minor 431 bp product (indicated by ↔ in Figure 1E) that represents a ZFP100 transcript variant lacking exon 2 completely was detected in cells treated with and without HU. The products of GAPDH mRNA and β-tubulin mRNA were of 1017 and 679 bp in length, respectively, corresponding to mRNAs containing all known exons, as confirmed by sequence analysis (Figure 1E). The relative proportions of all these mRNAs were not significantly affected by the treatment with HU. This indicates that alternative splicing is not a general consequence occurring under replication stress conditions and does not even apply to all factors specifically involved in the control of histone gene expression.

### HBP/SLBP mRNA alternative splicing is linked to replication stress conditions and is abrogated by treatment with caffeine

It is generally assumed that the full length HBP/SLBP protein encoded by the major transcript found in S-phase cells is the form involved in the regulation of histone gene expression. The observation that HBP/SLBPΔE2, HBP/SLBPΔE3 and HBP/SLBPΔE2+ΔE3 splice variants accumulate in HU-treated cells indicated that replication stress conditions may enhance alternative splicing of HBP/SLBP. Therefore we next examined in more detail the correlation between the induction of replication stress conditions and the effect that this has on the process of HBP/SLBP mRNA splicing.

**Figure 2:**
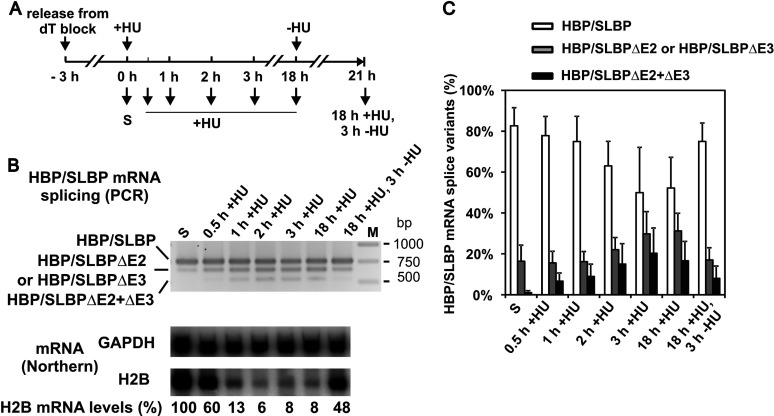
Alternative splicing of HBP/SLBP transcripts is linked to replication stress (A) A depiction of the experimental timeline. HeLa cells were synchronised by dT block and released into S phase. After 3 h, HU was added to cells for 18 h, then the HU was removed and the cells were incubated for a further 3 h. Time points when samples were taken for analysis are indicated. (B) Analysis of histone gene expression and HBP/SLBP mRNA alternative splicing. Top panel: analysis of HBP/SLBP mRNA splicing at indicated time points by reverse transcription followed by PCR. Relevant size standard fragments are indicated (M). Bottom panels: Northern blot analysis of histone H2B mRNA levels at the indicated time points. The blot was also probed for GAPDH mRNA. Histone mRNA levels were normalised with respect to GAPDH mRNA levels, and histone mRNA levels in the absence of HU (S) were defined as 100%. (C) The graph summarises the quantification of the HBP/SLBP splice isoforms (expressed as % of total HBP/SLBP transcripts detected) from four independent analyses of HBP/SLBP mRNA splicing. Shown are the means±S.D..

HeLa cells were synchronised by double thymidine block, then released into S phase, and subsequently treated with/without HU. In addition, HU was removed from the treated cells after an extended exposure of 18 h, to allow for the resumption of DNA replication (Figure 2A). An analysis of histone H2B mRNA levels by Northern blotting showed that as expected histone H2B mRNA disappeared rapidly after the addition of HU (Figure 2B) [28]. After 1 h, histone H2B mRNA levels were reduced to 13% of the level prior to addition of HU and remained low until the HU was removed. Approximately 3 h after removal of HU, histone mRNA levels had recovered to 48%. This typical response of histone H2B mRNA levels indicates that inhibition of DNA replication leads to the inhibition of histone gene expression and that removal of the inhibitor allows for restarting of DNA replication and thus histone gene expression.

In parallel, we tested for HBP/SLBP mRNA alternative splicing. We mainly detected full length transcripts in S-phase cells and a small amount of HBP/SLBPΔE3 or HBP/SLBPΔE2 transcripts (Figure 2B). Extrapolating from the bar chart summarizing the data from four independent experiments (Figure 2C), the amount of these alternative splice variants increased approximately 2-fold from 16% in S-phase cells to 31% after 18 h exposure to HU and, intriguingly, after removal of HU, their levels decreased again approximately 2-fold to 17%. Strikingly, the HBP/SLBPΔE2+ΔE3 splice variant was undetectable in S-phase cells, and increased over time in the presence of HU, reaching a peak (20%) after 3 h of exposure. It decreased to nearly undetectable levels (8%) after removal of HU. Concurrently, the proportion of full-length HBP/SLBP transcripts decreased from 83% in S-phase cells to 50% after prolonged exposure to HU, and increased again to 75% upon removal of HU. These findings indicate that replication stress conditions do not silence the transcription of the HBP/SLBP gene, but affect the splicing process. In Figure 2(C), it can been seen that there are some minor differences between experiments with regards to the proportion of HBP/SLBP mRNAs being subject to alternative splicing. However, these results confirm that alternatively spliced forms lacking exons 2 and/or 3 accumulate with time in the presence of HU, and disappear after removal of HU. In summary, this indicates that the alternative splicing of HBP/SLBP mRNA is influenced by DNA replication and clearly increases under DNA replication stress conditions.

**Figure 3:**
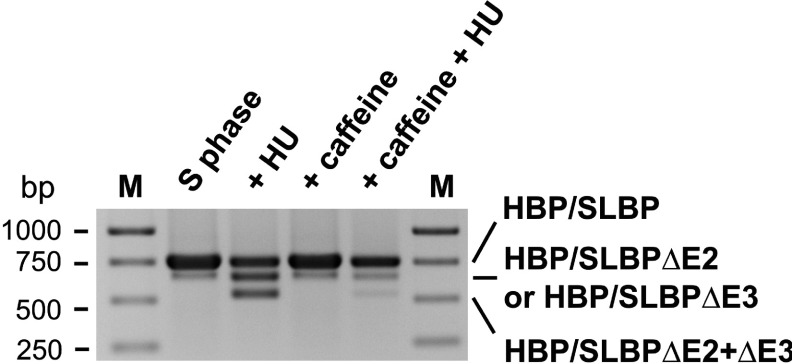
Replication stress-induced alternative splicing of HBP/SLBP mRNA is sensitive to caffeine HeLa cells synchronised by dT block were treated with/without 5 mM caffeine 3 h after release into S phase. After 1 h further incubation, caffeine treated as well as untreated HeLa cells were treated with HU for a further 3 h. Control cells (S phase) were left untreated. HBP/SLBP mRNA splicing was analysed as described. M indicates the molecular markers.

We wanted to test whether alternative splicing of HBP/SLBP mRNA in replication stress conditions may be dependent on checkpoint function. We and others have demonstrated previously that treatment with HU causes cell cycle checkpoint activation leading to the rapid decay of histone mRNA by a mechanism that is not well understood [27,28]. To examine the possibility that HBP/SLBP mRNA splicing is similarly dependent on checkpoint function we treated cells with caffeine, an inhibitor of the checkpoint kinases ATR and ATM. Although caffeine has multiple pharmacological targets, it inhibits the ATM and ATR kinases *in vitro* at concentrations close to those required to sensitize cells to killing by DNA-damaging agents [36,37]. In addition, caffeine blocks the radiation-induced ATM-dependent phosphorylation of Chk2 on Thr^68^ [37]. Pre-treatment of cells with 5 mM caffeine prior to the addition of HU led to a significant reduction of HBP/SLBP mRNA alternative splicing compared with splicing in cells treated with HU only (Figure 3). This strongly suggests HBP/SLBP mRNA splicing being controlled by caffeine-sensitive checkpoint kinases; however, the manner of this control is unclear. It is possible that the kinases control the activity of the splicing machinery, or act on other as yet unidentified downstream factors.

### Alternative splicing produces HBP/SLBP proteins lacking elements important for histone mRNA stability control and translation

HBP/SLBP protein binds the conserved histone mRNA hairpin element with high sequence specificity and affinity [38]. We and others have previously shown that the RBD (RNA-binding domain) encoded by exons 5 and 6 is sufficient for RNA binding [34,39,40]. The RBD and a domain located immediately to the C-terminal side of the RBD important for histone mRNA processing are not affected by alternative splicing [34,39,41]. Residues in the N- and C-terminal regions flanking the RBD of HBP/SLBP contribute to the high sequence – and structure – specificity of RNA binding. However, removing these regions does not actually abolish RNA binding nor does it allow for binding to RNA with any major deviations from the hairpin RNA consensus sequence [34,40].

**Figure 4:**
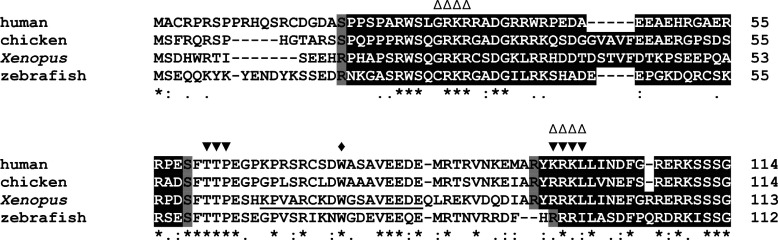
The exon structure and sequence elements in HBP/SLBP exons 1–4 are conserved in vertebrates (A). Alignment of N-terminal regions of human, chicken and zebrafish HBP/SLBP and *Xenopus* SLBP1. Identical and similar amino acids are indicated with stars and dots, respectively. Regions encoded by exons 2 and 4 are boxed in black, with exon 3 in between. Amino acids boxed in grey are encoded by triplets spanning exon junctions. Residues of *Xenopus* SLBP1 important for translation are underlined [21,23], and residues important for human HBP/SLBP protein stability (▼) and cellular localization (∆) are indicated [11–13]. Also indicated is Trp^75^ essential for the interaction of HBP/SLBP protein with CTIF (◆) [25].

Exons 2 and 3 encode elements in *Xenopus* SLBP1 known to be important for histone mRNA translation, and elements in human HBP/SLBP essential for its cellular localization and stability (Figure 4). The region Lys^65^–Gln^82^ of *Xenopus* SLBP1 is important for translation and the interaction with SLIP1, a factor specifically involved in translation of histone mRNA [21,22,24]. The alignment of the N-terminal sequences of human, chicken and zebrafish HBP/SLBP and *Xenopus* SLBP1 indicates that this region encoded by exon 3 is conserved in chicken, zebrafish and human proteins (Figure 4). We have shown previously that *Xenopus* SLBP1 lacking the exon 3 region is unable to stimulate cap-dependent translation initiation [22]. Furthermore, replacing Trp^75^ with Ala in the human protein disrupts an interaction with the CTIF [25]. CTIF localises to the nuclear envelope and binds to the CBP80 in the mRNA being exported, and is instrumental in recruiting translation initiation factors and the 40S ribosomal subunit to mRNA [26]. Thus, there is evidence implicating this HBP/SLBP region in a pioneer round of translation mediated by cap-binding proteins as well as eIF4E-dependent translation initiation and indicating that HBP/SLBP lacking this region is unable to fulfil this function. Interestingly, overexpression of HBP/SLBP with Ala^75^ also reduces the efficiency of replication stress-induced histone mRNA decay. It has been reported earlier that histone mRNA translation and mRNA decay are linked and this observation points towards a key role for HBP/SLBP in these processes [25,42].

The sequences Thr^61^Thr^62^Pro^63^ and Lys^96^Arg^97^Lys^98^Leu^99^ are important for the degradation of human HBP/SLBP upon exit from S phase. Thr^61^Thr^62^Pro^63^ encoded by exon 3 is a target for cyclin-dependent phosphorylation, and Lys^96^Arg^97^Lys^98^Leu^99^ encoded by exon 4 is a consensus cyclin binding site [12,13]. Cyclin-mediated phosphorylation of two threonine residues in the Thr^61^Thr^62^Pro^63^ sequence at the end of S phase is required for proteasome-mediated degradation of HBP/SLBP [10,12]. The sequence Lys^96^Arg^97^Lys^98^Leu^99^ has also been implicated in the nuclear localization of HBP/SLBP [13]. This element, together with the sequence Arg^31^Lys^32^Arg^33^Arg^34^ in exon 2 and a third sequence element close to the C-terminus Lys^246^Val^247^Arg^248^His^249^, is involved in nuclear import of HBP/SLBP mediated by Importin α/Importin β [13]. These elements are essentially conserved between human, chicken and zebrafish HBP/SLBP and *Xenopus* SLBP1. Finally, in addition to phosphorylation of Thr^61^ and Thr^62^, phosphorylation of Ser^20^ and Ser^23^ encoded by exon 1 and 2 has recently been connected to the destabilization of human HBP/SLBP by ubiquitination [11]. Despite the apparent importance of this region it is poorly conserved.

### Alternative splicing produces HBP/SLBP isoforms with different cellular localizations

We decided to investigate the effect of alternative splicing on the cellular localization of HBP/SLBP. We included in this analysis all the isoforms encoded by the splice variants detected, namely HBP/SLBP, HBP/SLBPΔE2, HBP/SLBPΔE3 and HBP/SLBPΔE2+ΔE3. The subcellular localization of HBP/SLBP has previously been examined by immunofluorescence using either antibodies raised against human HBP/SLBP, or expression of tagged HBP/SLBP from a transgene [13,43]. This has led to the view that HBP/SLBP is localised to both the nucleus and cytoplasm during S phase.

**Figure 5:**
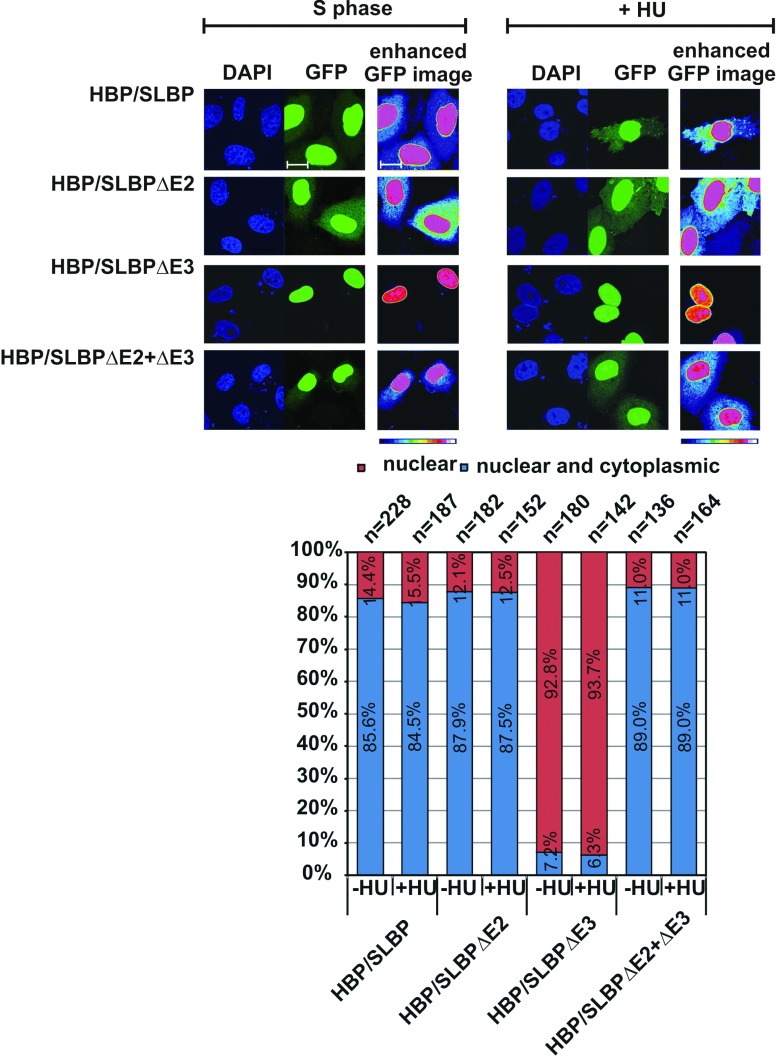
Alternative splicing alters the subcellular localization of HBP/SLBP S phase HeLa cells synchronised by dT block and expressing the indicated GFP–HBP/SLBP isoforms were prepared and analysed by confocal microscopy as described in Materials and Methods (S phase). For the treatment with HU, S-phase cells were supplemented with HU and incubated further for 1 h (+HU). Images of typical cells observed in each case are shown. DAPI staining was used to detect nuclei and protein localization was detected by GFP fluorescence. The subcellular localization of the GFP–HBP/SLBP isoform fusions were categorised as nuclear (nuclear localization of GFP–HBP isoform fusions with only background levels observed in the cytoplasm) or nuclear and cytoplasmic (nuclear localization of GFP–HBP isoform fusions and higher than background levels observed in the cytoplasm). The bar graph summarises the analysis of protein localization. n indicates the number of cells analysed. The percentages of cells in each category are represented as a proportion of stacked bars totalling 100%. GFP staining was enhanced using ImageJ software to detect low levels of cytoplasmic HBP/SLBP above background fluorescence. The scale bar is 20 μm.

To examine the effect that alternative splicing may have on the localization of the generated HBP/SLBP isoforms, we prepared constructs for the expression of the different HBP/SLBP isoforms with an N-terminal GFP tag. The constructs were transfected into HeLa cells and the cellular localization was analysed by confocal microscopy. Since HBP/SLBP is normally expressed in S phase, we synchronised HeLa cells by double thymidine block and specifically examined the localization of these proteins in S phase cells. Figure 5 shows that GFP-HBP/SLBP protein was detected in both the nucleus and cytoplasm of the majority of cells (85.6% nuclear and cytoplasmic staining). In light of previous observations by others, this was an anticipated result [13,43]. Similarly, GFP–HBP/SLBPΔE2 and GFP–HBP/SLBPΔE2+ΔE3 proteins were detected in both the nucleus and cytoplasm in the majority of cells (≥87% nuclear and cytoplasmic staining). On the other hand, GFP–HBP/SLBPΔE3 was predominantly detected in the nucleus (92.8%), and its cytoplasmic staining was weak and concentrated on regions close to the nucleus. We also examined whether the localization of the GFP-fusion proteins is dependent on histone mRNA as this has been reported to contribute to the cytoplasmic localization of HBP/SLBP [13]. We treated the cells with HU, which causes rapid degradation of histone mRNA (e.g. see Figure 2) and found that this did not fundamentally affect the subcellular localization of the fusion proteins (Figure 5). Together, this indicates that the localization we detected is a property of the various HBP/SLBP proteins independent of histone gene expression.

In conclusion, our results indicate that the cellular localization of HBP/SLBP isoforms observed here is an intrinsic property of these proteins. While HBP/SLBP, HBP/SLBPΔE2 and HBP/SLBPΔE2+ΔE3 localise to both the nucleus and cytoplasm, HBP/SLBPΔE3 localises predominantly to the nucleus.

## DISCUSSION

The histone mRNA-binding protein HBP/SLBP is a key regulator of the post-transcriptional control of histone gene expression and is instrumental in ensuring that histone protein synthesis only takes place in S phase, allowing for the packaging of newly replicated DNA. HBP/SLBP is a key component of the machinery processing histone mRNA 3′ ends and functions in cap-dependent translation initiation of histone mRNA. The expression of HBP/SLBP is cell cycle regulated whereby its protein synthesis is limited to S phase by an unclear mechanism controlling HBP/SLBP mRNA translation [9]. Here, we report the identification of alternative splice variants of human HBP/SLBP mRNA (Figure 1C), that lack exon 2 (HBP/SLBPΔE2) or exon 3 (HBP/SLBPΔE3) or both exons 2 and 3 (HBP/SLBPΔE2+ΔE3) and the detection of an additional smaller HBP/SLBP protein isoform (Figure 1D). Our data indicate that alternative splice variants are rare in S-phase cells but are enriched in HeLa cells subjected to replication stress conditions.

It is well known that under replication stress conditions, signalling involving checkpoint kinases leads to the arrest of the production of replication-dependent histone proteins at various different levels of their metabolism [27,28,44]. We found that alternative splicing of HBP/SLBP is specifically enhanced under replication stress and that notably it does not appear that replication stress conditions induced widespread changes to alternative splicing (Figures 1 and 2). We observed that levels of alternatively spliced HBP/SLBP transcripts decrease again after recovery from replication stress (Figure 2). This, together with the observation that alternative splicing is abrogated by the presence of the checkpoint kinase inhibitor caffeine (Figure 3), indicates that alternative splicing of HBP/SLBP is a physiological, checkpoint-mediated response that may contribute to the down-regulation of histone gene expression observed under replication stress conditions.

Our observations indicate that checkpoint kinases also act to increase the alternative splicing of HBP/SLBP mRNA, a key regulator of histone mRNA metabolism. The increase in alternatively spliced HBP/SLBP transcripts under replication stress conditions is likely to have a physiological meaning for the cell. It has been shown that under replication stress conditions, the histone mRNAs disappear whereas the HBP/SLBP protein levels remain unaffected [43]. Now with our results, it is clear that the expression of HBP/SLBP is linked to DNA replication in a previously unappreciated manner. Of course, this finding begs the question as to why exactly this happens and what function these alternative HBP/SLBP proteins might serve in cells exposed to replication stress conditions. Alternative splicing of exons 2 and/or 3 leads to the inclusion or omission of sequences important for HBP/SLBP localization and stability, and for the HBP/SLBP function in histone mRNA metabolism such as its role in histone mRNA translation (Figures 1 and 4) [12,13,22,23,34]. Removal of these sequences is expected to create HBP/SLBP isoforms with different properties.

It has been reported that HBP/SLBP localises predominantly to the nucleus at the beginning and end of S phase [13]. During S phase, it is also found in the cytoplasm. Using GFP-tagged HBP/SLBP isoforms we observed that alternative splicing radically affects the cellular localization of HBP/SLBP isoforms. Similar to endogenous HBP/SLBP, full-length GFP-tagged HBP/SLBP and the isoforms HBP/SLBPΔE2 and HBP/SLBPΔE2+ΔE3 localised to both the nucleus and cytoplasm (Figure 5). Exon 2 contains one of three elements previously identified as important for nuclear import mediated by Importin α/β, the others being located in exons 4 and 8 [13]. The observation that loss of the region encoded by exon 2 does not affect localization is in agreement with the observation that the omission of at least of two, but even better a lack of all three elements was required for a significant loss of nuclear localization [13]. In contrast, the HBP/SLBPΔE3 isoform localised predominantly to the nucleus, with only limited cytoplasmic staining. The reason for the reduction in cytoplasmic localization of HBP/SLBPΔE3 is not clear. Perhaps, the joining of exons 2 and 4 enhances the nuclear localization signal by bringing residues encoded by exon 2 such as Arg^55^Arg^56^ closer to Lys^96^Arg^97^Lys^98^Leu^99^. Alternatively, exon 3 may be important for nuclear export and loss of this sequence may result in retention of the HBP/SLBPΔE3 protein in the nucleus. The observation that HBP/SLBPΔE2+ΔE3 protein lacking exon 2 sequence in addition to exon 3 is found in the nucleus and cytoplasm suggests that there may be complex interactions between sequences in this region of the protein which are important for cellular localization.

The replication stress-induced production of a predominantly nuclear HBP/SLBP isoform may contribute to the observation by others that HBP/SLBP is localised in the nucleus in cells treated with HU [20,43]. This observation led to the proposal that co-export of HBP/SLBP with histone mRNA from the nucleus contributes to the cytoplasmic localization of HBP/SLBP observed during S phase and that relocation of HBP/SLBP into the nucleus under replication stress conditions is linked to the degradation of histone mRNA. In our hands, treatment with HU did not abolish the cytoplasmic localization of HBP/SLBP. It may be that analysis by confocal microscopy allows for the clearer detection of cytoplasmic HBP/SLBP, and, as HBP/SLBP is overexpressed we may detect the underlying subcellular distribution of HBP/SLBP independent of the presence of histone mRNA.

HBP/SLBP binds to histone mRNA and plays a role in all aspects of its metabolism, from RNA 3′ processing and possibly histone mRNA export in the nucleus to translation initiation and histone mRNA degradation in the cytoplasm. It promotes these processes by undergoing protein–protein interactions that promote the recruitment of factors involved in these processes. All of the HBP/SLBP isoforms still contain the well characterised residues necessary for the role of HBP/SLBP in histone pre-mRNA processing. For example, the residues necessary to interact with ZFP100 are present in all isoforms [14]. It has been proposed that HBP/SLBP acts in histone mRNA export [20] and an attractive hypothesis is that the HBP/SLBPΔE3 isoform inhibits export of histone mRNA from the nucleus since it is predominately localised to the nucleus (Figure 5). Therefore we tested the various HBP/SLBP isoforms in a nuclear export assay [45]. However, we failed to detect any significant activity of either the wild-type or the isoform versions (results not shown), and thus were unable to test this hypothesis. Furthermore, exon 3 in particular contains an element important for binding to CTIF, SLIP1, eIF3 and PAIP1 [22,25,46]. These factors are important for translation initiation and we have shown that loss of this region impedes any role of HBP/SLBP in translation [22,23]. In a recent report the interaction between zebrafish SLIP1 and the HBP/SLBP element important for translation (Figure 4), termed the SBM (SLIP1-binding motif) by the authors, has been characterised [47]. This motif has been found in two other proteins, the translation factor eIF3g and the nuclear export factor DBP5, and the binding of these proteins to SLIP1 was confirmed experimentally. SLIP1 forms homodimers, and the authors propose that these homodimers may recruit factors such as eIF3g or DBP5 to histone mRNA via an interaction with HBP/SLBP. It was also proposed that the recruitment of CTIF to histone mRNA is in the form of a SLIP1–CTIF heterodimer, although other evidence indicates that the interaction between HBP/SLBP and CTIF can be SLIP1 independent [25]. Therefore producing an isoform of HBP/SLBP that is unable to interact with key translation factors, HBP/SLBPΔE3, may serve as a further level of regulation to repress the production of histone proteins under replication stress conditions.

It should be noted that there are also known interactions which have not yet been mapped. For instance, HBP/SLBP interacts with Pin1, which facilitates RNA binding and also the ultimate degradation of HBP/SLBP itself. HBP/SLBP has also been found to interact with UPF1, a factor involved in mRNA decay [11,27]. It is possible that alternative splicing leads to the production of HBP/SLBP proteins that are unable to make critical interactions with other proteins and thus interferes with the function of HBP/SLBP in various parts of histone mRNA metabolism. This might include the synthesis of HBP/SLBP isoforms lacking elements for its normal regulation or localization, thus making it unable to participate in histone mRNA regulation. Alternatively, it may also create proteins that are able to undergo new, not yet identified interactions that do not normally occur in undisturbed S-phase cells. It would have been interesting to explore the effects of the absence of these sequences on the structure of HBP/SLBP; however, to date no structure is available for full-length human HBP/SLBP.

In conclusion, our findings indicate that HBP/SLBP activity is not only controlled by cell-cycle regulated expression but also by alternative splicing of HBP/SLBP mRNA. This leads to the production of significant levels of transcripts lacking sequences coding for elements involved in the function of HBP/SLBP protein in the control of histone gene expression. We propose that this is a newly identified layer of the control of histone gene expression acting during S phase. Our results indicate that this involves components of the checkpoint response. It will be interesting to explore the molecular mechanisms that control HBP/SLBP mRNA splicing and to investigate the functions of the HBP/SLBP isoforms produced under replication stress conditions.
